# Supplementary material for: The Glycan Ectodomain of SARS-CoV-2 Spike Protein Modulates Cytokine Production and Expression of CD206 Mannose Receptor in PBMC Cultures of Pre-COVID-19 Healthy Subjects
Source: Viruses. 2024 Mar 24;16(4):497. doi: 10.3390/v16040497 (PMC11054381; doi:10.3390/v16040497)
Supplement: Supplementary file 1 [file viruses-16-00497-s001.zip › viruses-2848506-supplementary.pdf]

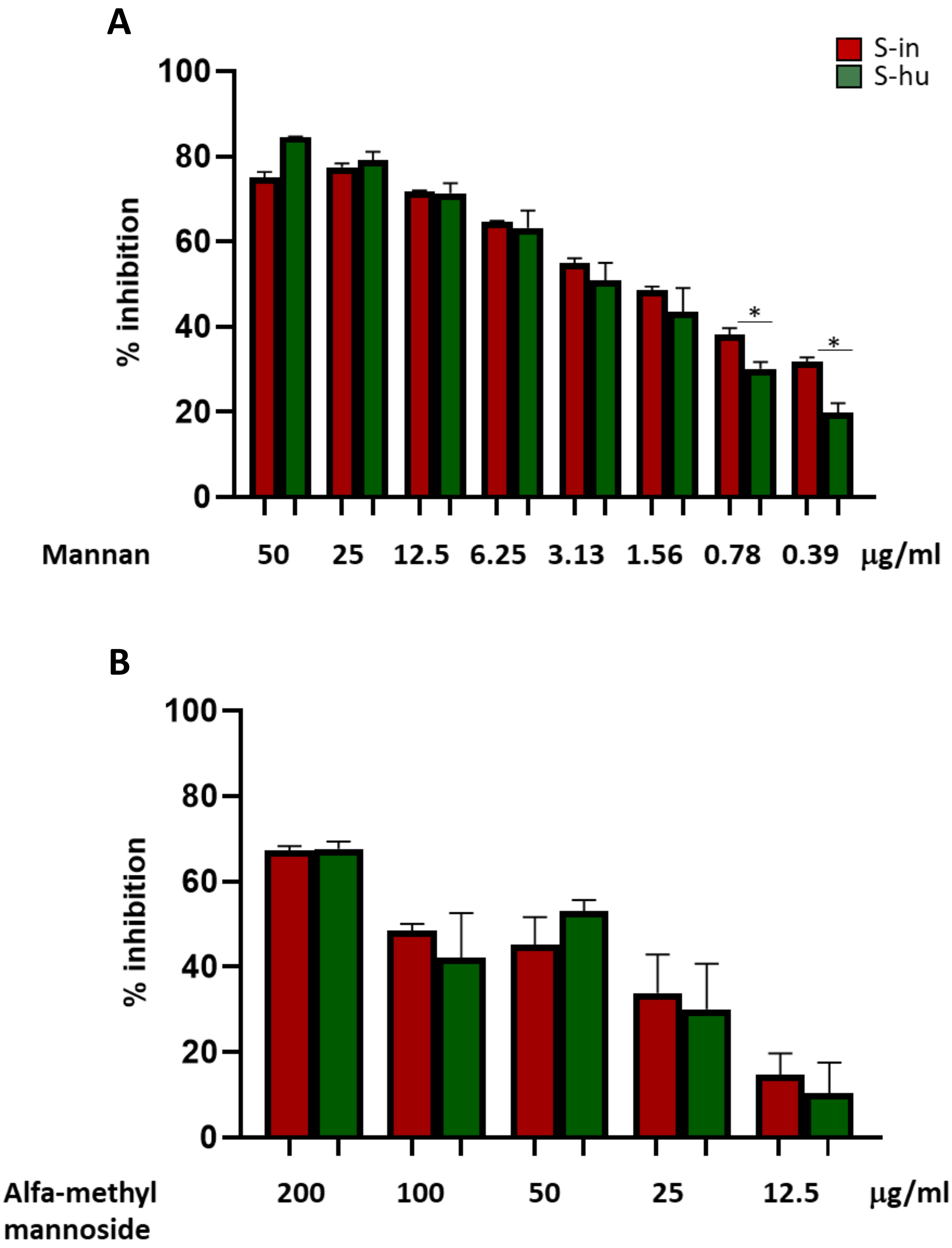

Figure S1. Mannan and alpha-methyl-mannoside inhibition of Spike (S) proteins and Con A binding. S-in or S-hu protein (0.75 µg/ml) were coated on a microplate and HRP-Con A (0.0125 µg/ml) binding was measured by optical density using an in-house ELISA assay. Scalar concentrations of saccharides were incubated together with HRP-Con A directly on the S protein coated microplates. Data indicated the percent of inhibition of scalar concentrations of mannan (top) or alpha-methyl-mannoside (bottom) and are reported as mean ± sd of two independent experiments run in duplicate. Statistical analysis: one-way ANOVA with Tukey's multiple comparisons post hoc test. *$p < 0.05$; **$p < 0.01$; ***$p < 0.001$; ****$p < 0.0001$.
Related to Figure 1

Supplementary Figure S1

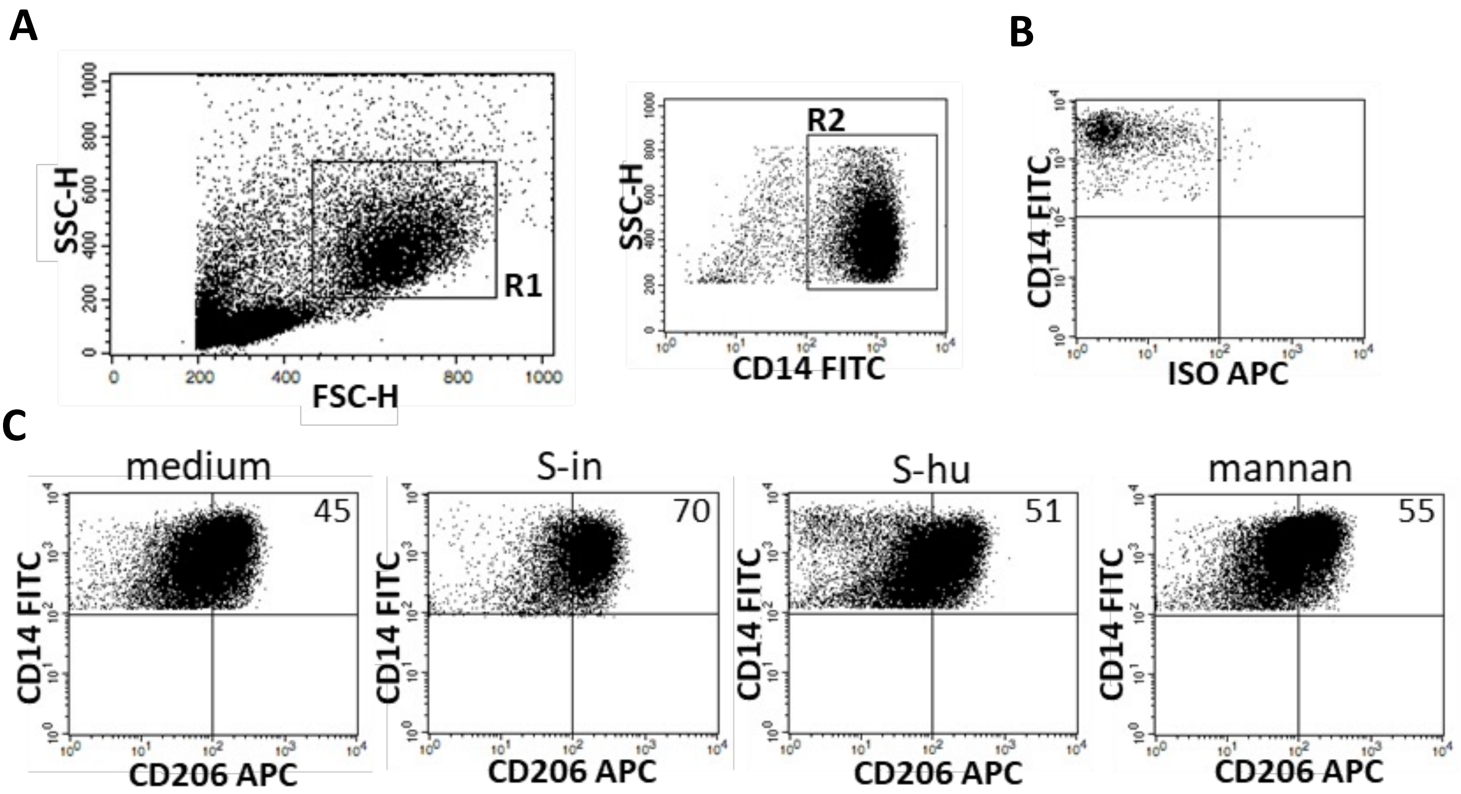

Figure S2. CD206 surface expression on CD14+ cells of PBMC stimulated with S-in, S-hu or mannan for the last 30 min of culture. (A) Gating strategy for flow cytometry analysis of CD206 expression on CD14+ macrophages. Representative dot plots showing gate on monocytes (R1) ad on CD14+ cells (R2) gated in R1. **(B)** Representative dot plot of CD14+ macrophage (gated in R2) versus Isotype APC. (**C)** Representative dot plots of CD14+ macrophages gated on R2 and positive for CD206 marker. Numbers reported in top right quadrants represent percentages of CD14/CD206 double-positive cells of PBMC cultured for 72 h and stimulated the last 30 min of culture with S-in 5 ug/ml, S-hu 5 ug/ ml or mannan 10 ug/ml. Control cells cultured only in medium are also show.

Related to Figure 7

Supplementary Figure S2
